# Safety, efficacy, and compliance of moderate-to-high dose eptinezumab and erenumab in chronic migraine patients with medication-overuse headache: an updated systematic review and meta-analysis

**DOI:** 10.1186/s10194-025-02047-7

**Published:** 2025-05-06

**Authors:** Nhan Nguyen, Vinh Ho Quang Tri, Vy Nguyen Ngoc Dan, Nghi Bao Tran, Laszlo Olah, Mate Heja

**Affiliations:** 1 Faculty of Medicine, University of Debrecen, Debrecen, Hungary; 2 Department of Neurology, Faculty of Medicine, University of Debrecen, Debrecen, Hungary; 3 Faculty of Medicine, University of Melbourne, Melbourne, Australia

**Keywords:** CM with MOH, Eptinezumab, Erenumab, MOH remission, ≥ 50% reduction in MMDs, Tolerability

## Abstract

**Background:**

The use of monoclonal antibodies targeting Calcitonin Gene-Related Peptide (CGRP) is an established treatment for chronic migraine (CM). However, its efficacy in CM patients with medication overuse headache (MOH) remains underexplored, and data on the safety and patient compliance of standard-to-high doses, especially Eptinezumab and Erenumab, over at least three months are limited.

**Objective:**

This study aims to evaluate the efficacy and safety of anti-CGRP therapy (Eptinezumab and Erenumab) in CM and MOH patients. Specifically, it assesses changes in monthly migraine days (MMDs) after 12 weeks, risk of treatment-emergent adverse events (TEAEs) leading to discontinuation, serious TEAEs, common adverse effects, and MOH remission at 6 months.

**Methods:**

A systematic search of PubMed, Cochrane, and Scopus databases identified randomized controlled trials (RCTs) evaluating standard or high dose anti-CGRP therapy in CM patients strictly with MOH. Studies included were required to report a ≥ 50% reduction in MMDs after ≥ 12 weeks, serious TEAEs, TEAEs leading to discontinuation, common adverse events, and MOH remission at 6 months. Heterogeneity was assessed using I² statistics and a random-effects model.

**Results:**

Three RCTs with 769 patients receiving standard-to-high dose anti-CGRP monoclonal antibodies (Eptinezumab and Erenumab) for ≥ 12 weeks were included. Anti-CGRP therapy significantly increased the likelihood of a ≥ 50% reduction in MMDs compared to placebo (OR: 2.43; 95% CI: 1.68–3.51; *p* < 0.00001). No substantial differences were found in TEAEs leading to discontinuation, nasopharyngitis, upper respiratory tract infections, or serious TEAEs between the anti-CGRP and placebo groups. The likelihood of MOH remission was approximately double in the anti-CGRP group (OR: 1.97; 95% CI: 1.40–2.78; *p* = 0.0001).

**Conclusion:**

Standard-to-high dose anti-CGRP therapies (eptinezumab, erenumab) effectively reduce monthly migraine days and improve MOH remission rates with minimal adverse effects, showing good tolerability in CM patients with MOH.

**Supplementary Information:**

The online version contains supplementary material available at 10.1186/s10194-025-02047-7.

## Introduction & objectives

Medication-overuse headache (MOH) is a common and disabling secondary headache disorder, as classified by the International Classification of Headache Disorders (ICHD-3) [1]. It results from the excessive use of acute headache treatments, such as simple or combination analgesics, triptans, and opioids. This overuse often leads to a worsening of existing headaches or the emergence of a new headache type. MOH affects individuals with underlying primary headache conditions, like chronic migraine (CM) or chronic tension-type headache, who experience frequent headaches (15 or more days per month) over at least three months while overusing acute headache medications [2]. Overuse is defined as using simple analgesics on 15 or more days per month or triptans, ergots, or opioids on 10 or more days per month.

The prevalence of MOH globally is estimated at 1–2% of patients with migraine, with variability depending on regional and methodological differences [3, 4]. Women are disproportionately affected, likely due to the higher occurrence of migraines and tension-type headaches in females [5]. Beyond personal suffering and disability, MOH imposes a significant global economic burden [6]. Indirect costs, including lost productivity and absenteeism, account for roughly 92% of the total economic impact [7]. MOH is also linked to psychiatric conditions such as depression and anxiety, further diminishing patients’ quality of life8. Those with MOH experience increased healthcare utilization, reduced efficacy of preventive treatments, and more severe headache symptoms, including higher frequency, intensity, and duration [8, 9]. However, reducing or discontinuing acute headache medications, coupled with preventive therapies targeting the primary headache disorder, often leads to symptom improvement [10].

Monoclonal antibodies (mAbs) targeting Calcitonin Gene-Related Peptide (CGRP) have demonstrated effectiveness in preventing migraine attacks in patients with chronic or episodic migraine (EM). The European Headache Federation recommends anti-CGRP therapies for migraine prevention, citing their long-term efficacy and safety, which align with the pathophysiology of migraine [11]. CGRP plays a pivotal role in migraine mechanisms, with its widespread expression in the central nervous system (CNS). Studies since 1990 have linked elevated CGRP levels to migraine attacks, which can be alleviated with treatments like sumatriptan [12]. Additional research has shown that CGRP levels are elevated in the cerebrospinal fluid (CSF) and jugular blood of chronic migraine patients, while intravenous CGRP can induce migraines experimentally [13].

The therapeutic action of anti-CGRP therapies appears to be peripheral, primarily targeting the trigemino-vascular system, including the meninges, trigeminal ganglion (TG), and nerve fibers. Upon activation, trigeminal fibers release CGRP, leading to vasodilation, neurogenic inflammation, and peripheral sensitization. These processes may contribute to the escalation of CGRP release, triggering inflammatory loops and nociceptive sensitization. In the spinal trigeminal nucleus, CGRP modulates glutamatergic signaling, potentially causing central sensitization and the perception of pain [14].

While the role of CGRP in migraine pathophysiology and the efficacy of anti-CGRP therapies in CM and EM is well-documented, their application for CM patients with MOH remains underexplored. Recent randomized controlled trials (RCTs) [15, 16, 17] and prior meta-analyses [18, 19] provide emerging evidence for efficacy of anti-CGRP therapy in patients with dual diagnosis of CM and MOH. However, less is known about the safety and effectiveness of high doses of anti-CGRP therapies in this specific subgroup, which warrants further investigation to address this discrepancies in management of coexisting CM and MOH.

## Materials & methods

This systematic review and meta-analysis were performed and reported under the Cochrane Collaboration Handbook for Systematic Review of Interventions and the Preferred Reporting Items for Systematic Reviews and Meta-Analysis (PRISMA) Statement guidelines [20, 21].

### Eligibility criteria

The Inclusion in this meta-analysis was restricted to studies that met all the following criteria: (1) RCTs; (2) comparing anti-CGRP monoclonal antibodies to placebo; (3) enrolling CM patients with MOH; (4) with follow up at least 3 months. In addition, studies were included if they reported any of the clinical outcomes of interest (Outcomes). We defined standard-to-high-dose anti-CGRP therapy as eptinezumab 100 mg/month or higher; fremanezumab 225 mg/month or higher; galcanezumab 120 mg/month or higher; and erenumab 70 mg/month or higher. For this meta-analysis, we focused on RCTs evaluating erenumab and eptinezumab in patients with MOH. These two monoclonal antibodies were selected based on the availability of detailed MOH-specific subgroup data, which was not as clearly reported for other CGRP-targeting mAbs. Additionally, differences in their mechanisms of action—erenumab targeting the CGRP receptor and eptinezumab targeting the CGRP ligand—allow for meaningful comparisons in MOH management.

A minimum of 3-month follow-up was chosen based on the curiosity of long-term outcomes posed on patients who underwent the anti-CGRP therapy, with regard to both the safety and efficacy of treatment. We excluded studies with (1) no control group or control group without placebo, (2) only CM or EM without MOH, and (3) observational studies, (4) studies involving Medication Overuse (MO) or mixed MO with MOH without MOH subgroup analysis.

### Search strategies

We systematically searched PubMed, Scopus, and Cochrane Central Register of Controlled Trials from inception to December 2024, with the following search strategy: (“Chronic Migraine” OR “Migraine Disorders”) AND (“Medication Overuse Headache” OR “MOH”) AND (“Monoclonal Antibodies” OR “Calcitonin Gene-Related Peptide” OR “CGRP” OR “Erenumab” OR “Fremanezumab” OR “Galcanezumab” OR “Eptinezumab”) AND (Placebo) AND (randomized controlled trial[pt] OR controlled clinical trial[pt] OR clinical trials as topic[mesh: noexp] OR trial[ti] OR random*[tiab] OR placebo*[tiab]).

**Fig. 1:**
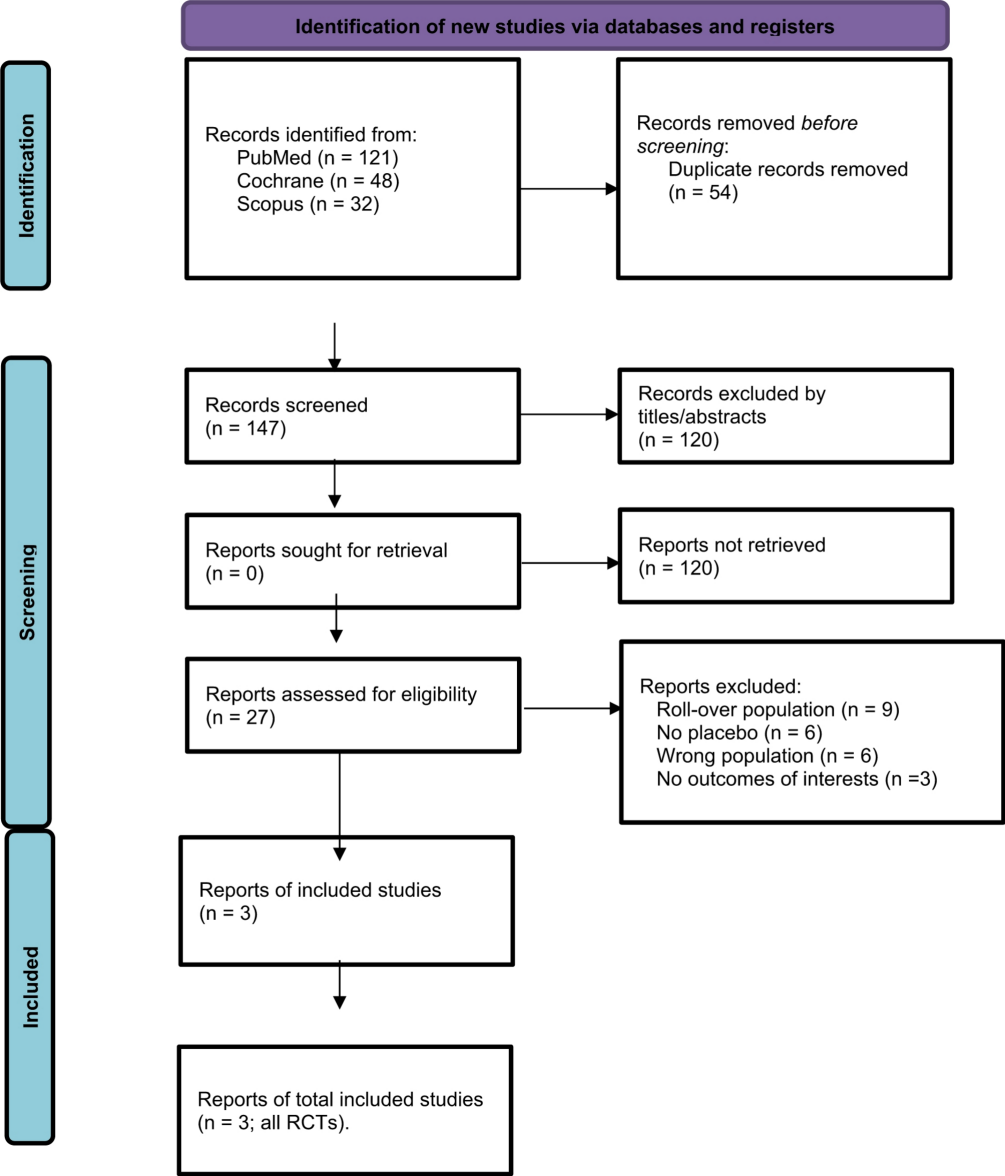
PRISMA 2020 flow diagram for updated systematic reviews which included searches of databases and registers only

**Fig. 2:**
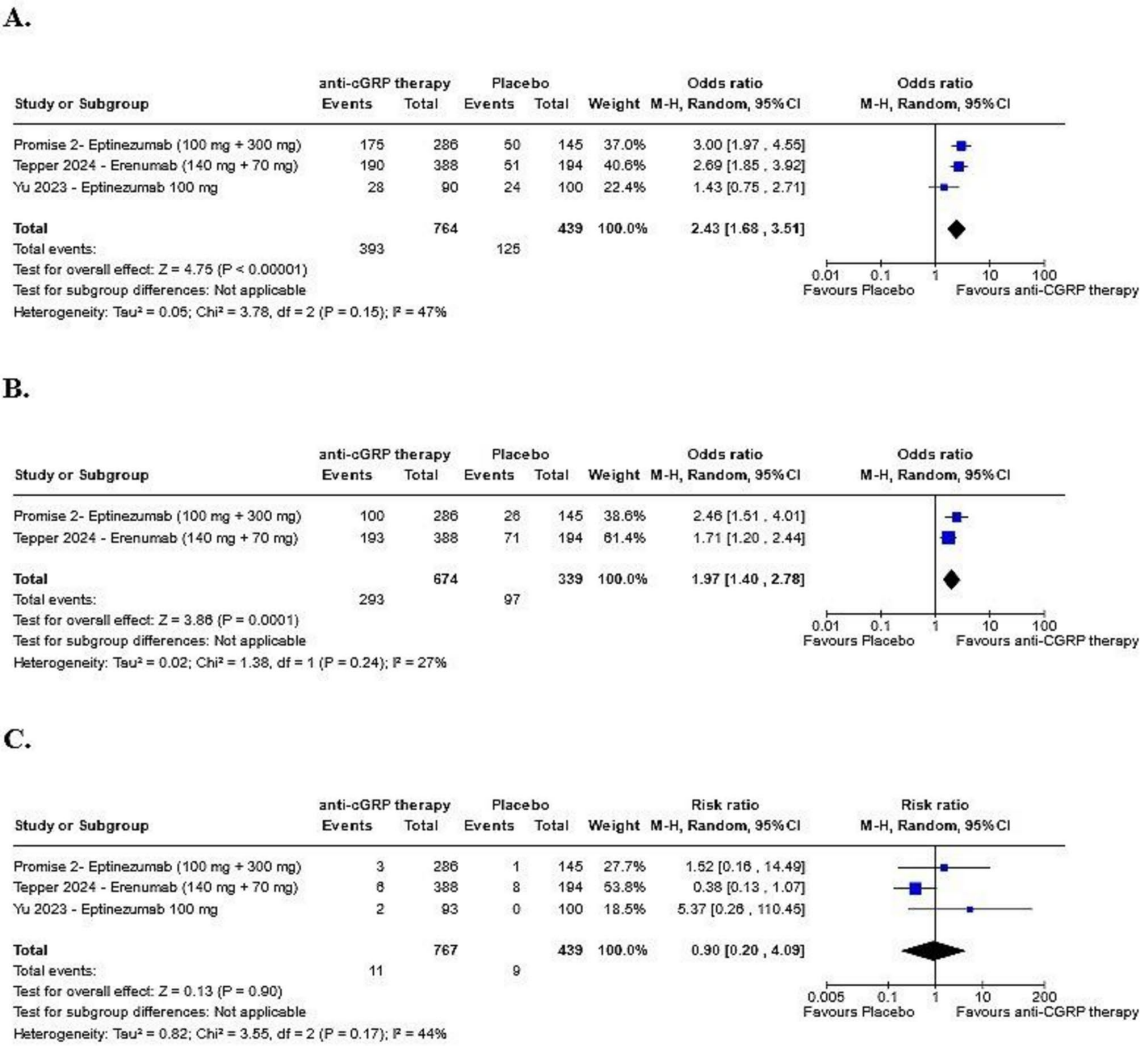
Safety and Efficacy Outcomes of Eptinezumab and Erenumab (combined standard and high dose therapy). **A**: At least a 50% reduction in MMDs from baseline with at least 12 weeks of treatment. **B**: Remission of MOH with at 6 months of treatment. **C**: Risk of TESAEs. All analyses used a *p* value < 0.05, with 95% confidence intervals exclude null value of 1 as statistical significant results

The preferences from all included studies, previous systematic reviews and meta-analyses were also searched manually for any additional studies. Two authors (N.N, and V.H.Q.T) independently extracted data following predefined search criteria and quality assessment. All the data in the forest plots were double-checked by the other 2 authors, Fig. 1. by N.T.B and Fig. 2 by V.N.N.D. MH and LO revised the manuscript critically for important content. The prospective meta-analysis protocol was registered on Prospero on May 12th, 2024; with ID (CRD42024618359).

### Outcomes

Efficacy outcomes included (1) at least a 50% reduction in monthly migraine days (MMDs), (2) MOH remission at 6 months, and treatment side effects including: (3) Nasopharyngitis, (4) Urinary Tract Infection (UTI); (5) Upper Respiratory Tract Infection (URTI); (6) Treatment-Emergent Adverse Events (TEAEs) leading to discontinuation or interruption; and (7) Treatment-Emergent Serious Adverse Events (TESAEs). MOH remission was defined as the absence of MOH, indicated by the mean monthly acute headache medication days (AHMD) of fewer than 10 days over a 3-month period, sustained at both 3 months and 6 months. MOH remission was defined as the absence of MOH, indicated by the mean monthly acute headache medication days (AHMD) of fewer than 10 days over a 3-month period, sustained at both 3 months and 6 months. Definitions of MMDs, TESAEs, and MOH remission are reported in the Supplementary file.

We selected dichotomous outcomes, such as the 50% response rate and MOH remission at 6 montsh, as the primary measures of treatment efficacy. This decision was based on their clinical relevance, widespread use in migraine research, and ease of comparability across trials. Continuous outcomes, such as the reduction in monthly migraine days, were not included due to variations in baseline migraine frequency, study designs, and reporting methods, which could introduce additional heterogeneity and limit data pooling.

### Quality assessment

Two independent authors completed the risk of bias assessment (N.N, and V.H.Q.T). For quality assessment of each randomized controlled trial (RCT), we utilized the Cochrane Collaboration’s risk of bias tool20. Each trial was evaluated across five domains—selection bias, performance bias, detection bias, attrition bias, and reporting bias—and assigned a risk level of high, low, or unclear. Disagreements were resolved through a consensus after discussing reasons for the discrepancy. If the disagreement was still not resolved, a third party (N.T.B) was involved in the discussions.

### Statistical analysis

Odds ratios (OR) with 95% confidence intervals were used to compare treatment effects for MOH categorical endpoints (MOH remission at 6 months, and at least 50% reduction in MMDs). For all safety outcomes, Risk ratios (RR) with 95% confidence intervals were utilized. Continuous outcomes were compared with standardized mean differences. Heterogeneity was assessed with I^2^ statistics and the Cochrane Q test; *p*-values < 0.10 and I^2^ > 25% were considered significant for heterogeneity. We used the DerSimonian and Laird random-effects model. Sensitivity analysis were also performed by removing each individual study from the outcome assessment. We used Review Manager 5.4 (Cochrane Center, The Cochrane Collaboration, Denmark) for statistical analysis.

### Sensitivity analysis

We performed a leave-one-out sensitivity analysis to ensure the results were not dependent on a single study. In addition, we conducted two subgroups of studies with (1) Eptinezumab 100 mg and Erenumab 70 mg (standard dose) treatment for all outcomes; and (2) only high dose anti-CGRP therapy subgroup including Erenumab 140 mg and Eptinezumab 300 mg.

### Differences from previous Meta-Analysis

Two recent meta-analyses [18, 19] on this topic included trials [24, 25, 26] that did not strictly adhere to the ICHD-3 criteria [1] for MOH, incorporating mixed populations of MO and MOH. This likely introduced heterogeneity and limited the applicability of their findings to specific MOH subgroups. In contrast, our meta-analysis addresses these limitations by exclusively including trials with strictly defined MOH populations or MOH subgroup analyses, thereby reducing bias and enhancing the specificity of results. Additionally, we strengthen the evidence base by incorporating a phase 4 trial to evaluate real-world safety outcomes, assessing treatment tolerability through TEAEs leading to discontinuation—key for understanding compliance in clinical settings—and conducting dosage-based subgroup analyses to provide clinicians with actionable insights for dose optimization. These advancements underscore the clinical relevance and reliability of our updated findings.

## Results

### Study selection and baseline characteristics

The initial search yielded 201 results. After the removal of duplicates by title and abstract, 27 studies remained and were fully reviewed based on the inclusion criteria. Of these, a total of 3 studies were included, comprising 865 patients from 3 RCTs. (Fig. 1).

A total of 769 patients received anti-CGRP therapy, while 439 patients were assigned to the placebo group. Two studies evaluated Eptinezumab [16, 17], and one study investigated Erenumab [15], with one study included standard dose only [17]. There was significant variability across studies in the treatment duration and follow-up periods, with the shortest follow-up being 12 weeks. We found no studies examining CM patients with MOH treated with galcanezumab or fremanezumab. MMDs were reported in all included studies, while MOH 6 months after anti-CGRP mAb treatment was published only in 2 of 3 studies [15, 16]. Mean age of participants was around.

40 years old across all studies. Additional details, including specific data, can be found in Table 1, and Supplementary.

**Table 1:** Baseline characteristics of included studies

Study	Design	Type of cGRPi	Doses (mg/ month)	Patients, *n* cGRPi/PBO	Male, % cGRPi/PBO	Age ^†^, cGRPi /PBO	Baseline MMDs ^†^, days cGRPi/PBO	Baseline MHD ^†^, days cGRPi/PBO	Baseline simple analgesic overuse, *n* (%) cGRPi/PBO	Baseline triptan/ergot/opioid overuse, *n* (%) cGRPi/PBO
Promise 2^16^	*RCT	Eptinezumab	Standard (100); High (300)	139; 147 / 145	13.7; 10.9 / 13.8	41.5 (11.36); 42.1 (10.16) / 40.7 (10.86)	16.7 (4.58) 16.7 (4.92) / 16.7 (4.43)	20.7 (3.00) 20.6 (3.28) / 20.7 (3.00)	26 (18.7) 19 (12.9) / 30 (20.7)	83 (59.7) 105 (71.4) / 88 (60.7)
Tepper 2024^15^	RCT	Erenumab	Standard (70); High (140)	195; 195 / 194	17.9; 14.9 / 19.6	43.2 (11.8); 43.5 (12.6) / 44.4 (12.6)	19.2 (4.6); 18.5 (4.6) / 18.6 (4.6)	20.8 (3.9); 20.7 (3.8) / 20.8 (3.9)	18 (9.2); 12 (6.2) / 19 (9.8)	132 (67.7); 140 (71.8) / 128 (66)
Yu 2023^17^	RCT	Eptinezumab	Only standard (100)	93 / 100	26.7 / 18	43.5 (4.9) / 44.5 (4.9)	19.5 (3.6) / 19.7 (3.8)	20.6 (2.9) / 20.9 (3.3)	N /A	N /A

^*^RCT: randomized controlled trial; PBO: Placebo; cGRPi: anti-Calcitonin Gene-Related Peptide monoclonal antibodies; ^†^mean (SD); MMD: Monthly Migraine Days; MHDs: Monthly Headache Days; n: number of events or participants

Definitions

1. MMDs: The total number of days in a calendar month during which a patient experiences a migraine attack meeting diagnostic criteria (e.g., pain severity, duration, associated symptoms such as nausea or sensitivity to light/sound)

2. MOH remission; ICHD−3 Criteria: Headache days reduced to < 15 days/month after discontinuing the overused medication for at least 2 months

3. TESAEs: A serious adverse event is defined as a medical event linked to a drug, device, or intervention that results in death, a life-threatening condition, hospitalization, significant disability, a congenital anomaly, or requires intervention to prevent permanent harm

4. MHDs: the total number of days in a calendar month where a patient experiences a headache, regardless of whether the headache fulfills the specific criteria for a migraine

### Pooled analysis of all included studies

#### Overall Anti-CGRP therapy efficacy (combined standard and high dose therapy)

**Fig. 3:**
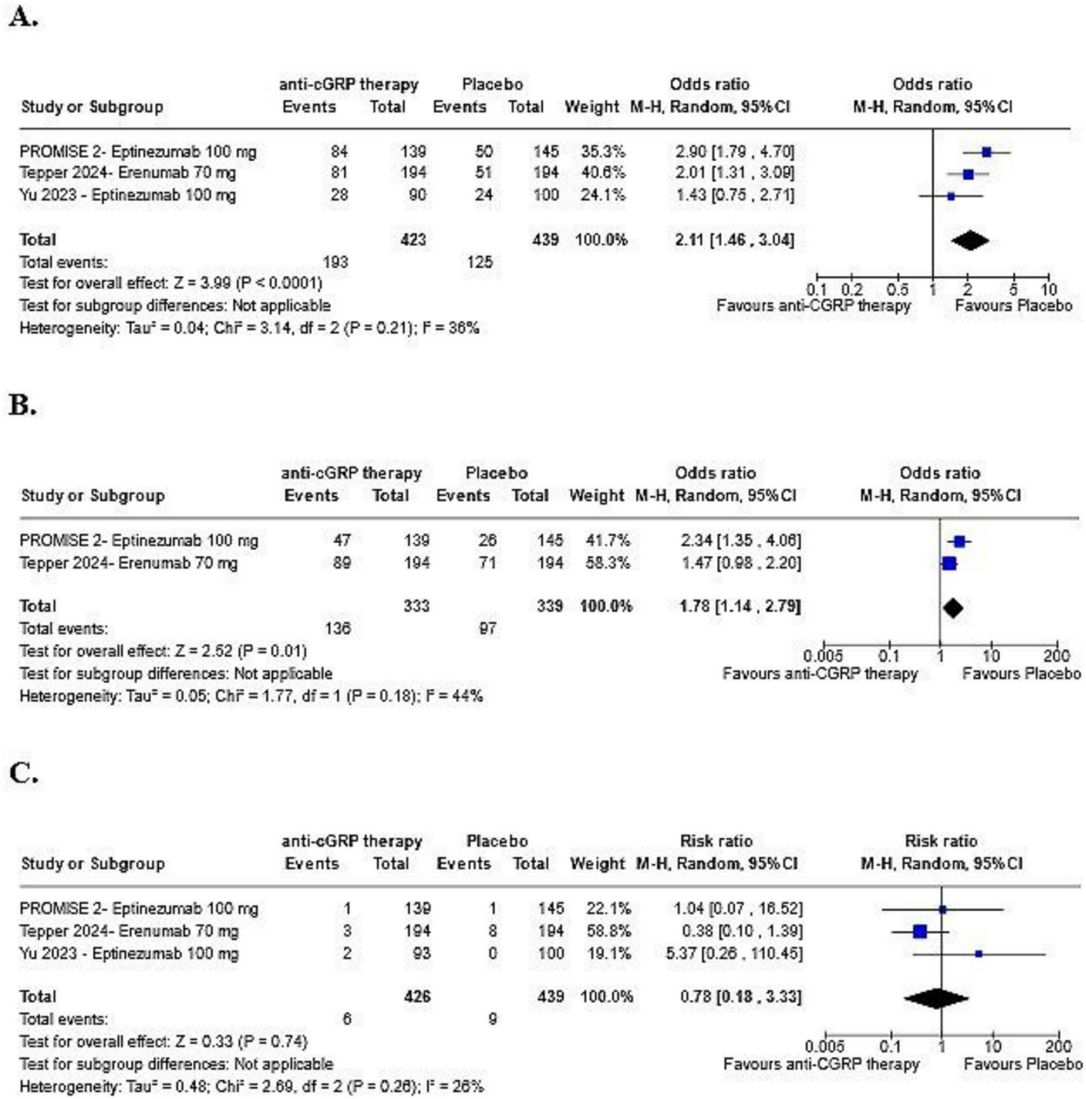
Safety and Efficacy Outcomes of Anti-CGRP Therapy (Eptinezumab 100 mg and Erenumab 70 mg, standard dose therapy). **A**: At least a 50% reduction in MMDs from baseline with at least 12 weeks of treatment. **B**: Remission of MOH with at 6 months of treatment. **C**: Risk of TESAEs. All analyses used a *p* value < 0.05, with 95% confidence intervals exclude null value of 1 as statistical significant results

**Fig. 4:**
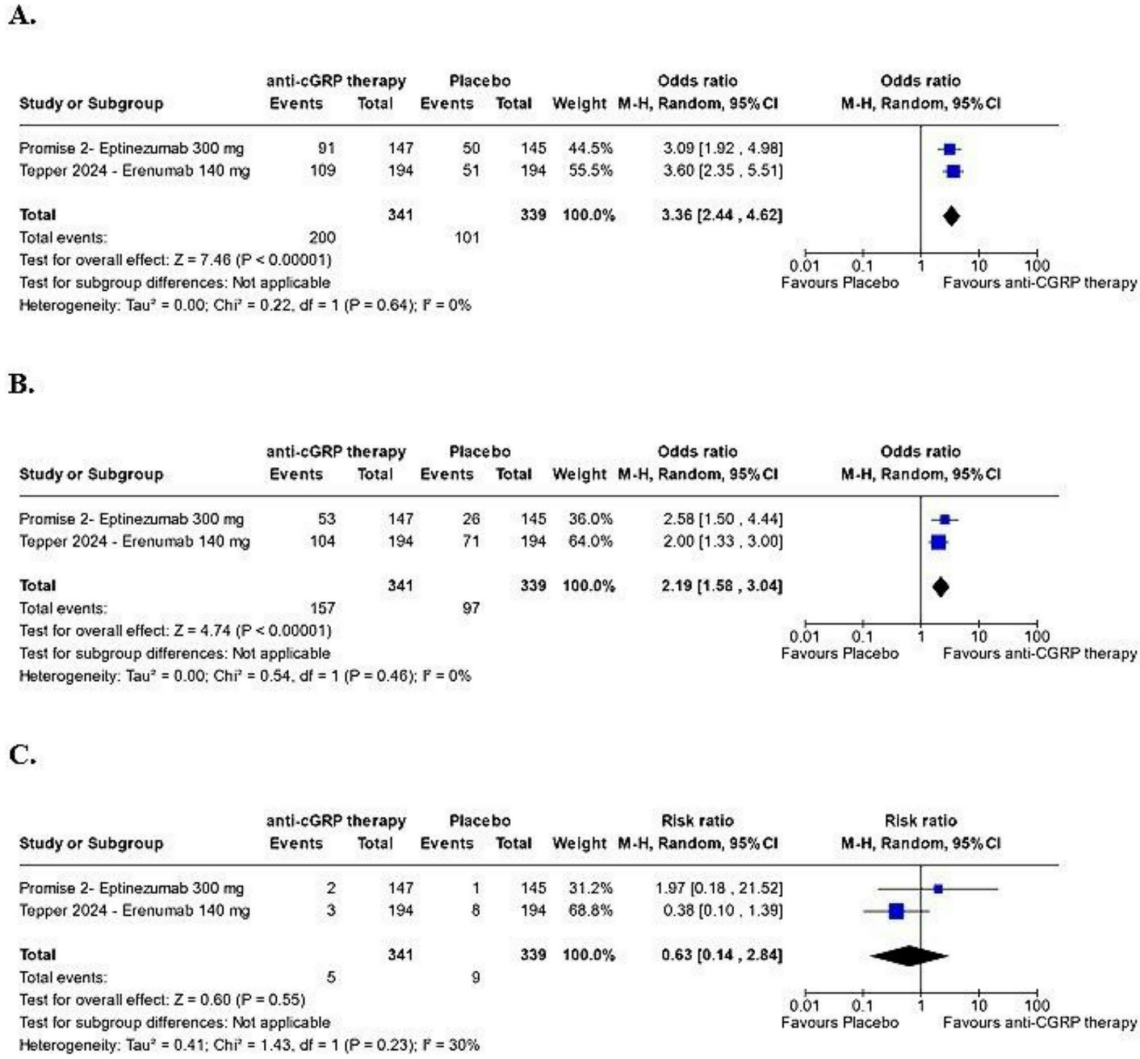
Safety and Efficacy Outcomes of Anti-CGRP Therapy (Eptinezumab 300 mg, Erenumab 140 mg, high dose therapy). **A**: At least a 50% reduction in MMDs from baseline with at least 12 weeks of treatment. **B**: Remission of MOH with at 6 months of treatment. **C**: Risk of TESAEs. All analyses used a *p* value < 0.05, with 95% confidence intervals exclude null value of 1 as statistical significant results

Figure 2 illustrates the overall efficacy of anti-CGRP therapies, MOH remission and safety outcomes. Patients who underwent anti-CGRP therapy were more than twice as likely to achieve at least a 50% reduction in monthly migraine days (MMDs) compared to the placebo group after at least 12 weeks (OR: 2.43; 95% CI: 1.68–3.51; *p* < 0.00001; I² = 47%; Fig. 2A). Moreover, the rate of MOH remission was nearly twice as high in the anti-CGRP group after at least 6 months of therapy compared to placebo (OR: 1.97; 95% CI: 1.40–2.78; *p* = 0.0001; I² = 27%; Fig. 2B). Safety outcomes, including (1) TESAEs (RR: 0.90; 95% CI: 0.20–4.09; *p* = 0.90; I² = 44%; Fig. 2C), (2) nasopharyngitis (RR: 1.86; 95% CI: 0.75–4.62; *p* = 0.18; I² = 36%; Fig. 2D), (3) upper respiratory tract infections (RR: 0.83; 95% CI: 0.45–1.54; *p* = 0.56; I² = 0%; Fig. 2E) and (4) discontinuation due to TEAEs (RR: 1.94; 95% CI: 0.79–4.81; *p* = 0.15; I² = 0%; Fig. 2F) did not differ significantly between the intervention and placebo groups. Figure 2D and F can be found in Supplementary.

#### Subgroup analysis: eptinezumab 100 mg and erenumab 70 mg (Standard Dose)

Figure 3. shows the subgroup analysis of standard dose therapies. Standard dose therapy demonstrated efficacy consistent with the combined analysis, significantly improving the likelihood of achieving a ≥ 50% reduction in MMDs compared to placebo (OR: 2.11; 95% CI: 1.46–3.04; *p* < 0.00001; I² = 36%; Fig. 3A). MOH remission rate at 6 months was higher for standard anti-CGRP treatment subgroup compared to placebo (OR: 1.78; 95% CI: 1.14–2.79; *p* = 0.01; I² = 44%; Fig. 3B). Safety outcomes showed no significant differences between the intervention and placebo groups for TESAEs (RR: 0.78; 95% CI: 0.18–3.33; *p* = 0.74; I² = 26%; Fig. 3C), nasopharyngitis (RR: 1.84; 95% CI: 0.48–7.05; *p* = 0.37; I² = 59%; Fig. 3D), upper respiratory tract infections (RR: 0.71; 95% CI: 0.33–1.54; *p* = 0.39; I² = 0%; Fig. 3E), or TEAE-related discontinuation (RR: 1.36; 95% CI: 0.47–3.92; *p* = 0.57; I² = 0%; Fig. 3F). Figure 3D and F can be found in Supplementary.

#### Subgroup analysis: High-Dose Anti-CGRP therapy (Eptinezumab 300 mg, erenumab 140 mg)

Figure 4. depicts the subgroup analysis comparing high-dose anti-CGRP therapies versus placebo. High-dose therapies were more effective than placebo as patients in the high-dose treatment subgroup were approximately three times more likely to achieve a ≥ 50% reduction in MMDs than patients in the placebo group (OR: 3.36; 95% CI: 2.44–4.62; *p* < 0.00001; I² = 0%; Fig. 4A). The rate of MOH remission at 6 months was twice in the high-dose anti-CGRP subgroup, compared to placebo (OR: 2.19; 95% CI: 1.58–3.04; *p* < 0.00001; I² = 0%; Fig. 4B). For TESAEs, there were no substantial differences between high dose anti-CGRP therapy and placebo, (RR: 0.63; 95% CI: 0.14–2.84; *p* = 0.55; I² = 30%; Fig. 4C). Safety outcomes remained similar between the high-dose intervention and placebo groups for all measured adverse events. These outcomes are reported in Fig. 4D and F (Supplementary).

### Sensitivity analysis

Due to heterogeneity, we performed a leave-one-out sensitivity analysis by iteratively removing one study at a time to ensure the results were not dependent on a single study. Overall, the removal of each study from the pooled analysis did not affect the safety and efficacy endpoints, except for nasopharyngitis in the standard-dose anti-CGRP subgroup. With the exclusion of the “PROMISE 2” study the risk of this outcome was higher for standard-dose anti-CGRP subgroup compared to placebo; which may be explained by the indirect calculation of events from percent reported in this study. (Supplementary)

### Quality assessment

The Risk of Bias (RoB 2) tool was used for quality assessment [22]. Most studies have some concerns about risk of bias as described in Supplementary Table 1 in the Supplementary Appendix. However, for Promise 2, we detected a high risk of bias due to result selections. This can be justified by the study nature, as this focused specifically on a subgroup of patients with MOH and CM in the original Promise 2, which may lead to the above risk.

## Discussion

In recent years, anti-CGRP mAbs have emerged as a promising therapeutic option for CM and MOH. This systematic review and meta-analysis of three RCTs involving 865 patients compared the efficacy and safety of moderate-to-high dose anti-CGRP therapy with placebo. The findings reveal significant insights into the potential benefits and limitations of this therapeutic approach.

The primary outcomes of this meta-analysis demonstrate that anti-CGRP therapy offers a substantial improvement in clinical outcomes for CM patients with MOH. Patients treated with anti-CGRP mAbs were more likely to achieve MOH remission at six months compared to those receiving a placebo. Additionally, the odds of experiencing at least a 50% reduction in MMDs from baseline were twice as high with anti-CGRP therapy. These results underscore the efficacy of anti-CGRP mAbs in reducing the frequency and severity of migraine days in this population. Safety and tolerability are critical considerations in evaluating any therapeutic intervention. This analysis found no significant differences between anti-CGRP therapy and placebo in the incidence of nasopharyngitis, upper respiratory tract infections, TESAEs, or discontinuation due to TEAEs.

Subgroup analyses provided further details, with Eptinezumab 100 mg and Erenumab 70 mg showing similar efficacy and safety outcomes as the overall analysis. Remarkably, the high-dose subgroup (Erenumab 140 mg and Eptinezumab 300 mg) demonstrated triple the odds of achieving a ≥ 50% reduction in MMDs compared to placebo, without an increased risk of adverse events. Although no direct comparison is available between standard-dose and high-dose anti-CGRP subgroups, meta-analysis data indicate that higher doses may offer enhanced efficacy without compromising safety in a long-term treatment course of at least 3 months, a crucial consideration for optimizing patient outcomes. Additionally, the potential dose-response relationship of CGRP-targeting monoclonal antibodies in MOH treatment remains uncertain. While higher doses of erenumab and eptinezumab appeared to show a trend toward greater reductions in headache frequency and medication overuse, the differences between standard and high doses were not consistently significant. Given that CGRP receptor occupancy is near-maximal at standard doses, further research is needed to determine whether increased dosing confers additional clinical benefit in MOH populations.

Furthermore, we analyzed the total study population for the MOH remission outcome rather than restricting it to the acute headache medication subgroup. This approach ensured consistency, as Tepper et al. (2024) [15] did not stratify their data similarly to PROMISE-2 [16]. Nevertheless, we have also reanalyzed the MOH remission outcome using the specific acute headache medication subgroup (Supplementary 1). Our results remained consistent, with no significant differences observed across both the high-dose and standard-dose subgroups.

Compared to last year’s meta-analysis [18], which claimed to assess both medication overuse (MO) and medication overuse headache (MOH), we found that only one of the four included studies—Marmura et al. (2021) [27], analyzing MOH subgroups from the PROMISE-2 trial—was relevant to MOH. PROMISE-2 was also included in our study [16]. Tepper et al. (2019) [25] and Silberstein et al. (2020) [26] focused on MO, while Dodick et al. (2020) [24] included mixed MO/MOH data without a distinct MOH subgroup. Thus, Sirilertmekasakul et al.‘s meta-analysis [18] primarily addressed MO rather than MOH. In contrast, our study exclusively analyzes the MOH subgroup in chronic migraine, ensuring a more precise evaluation of this population.

In addition to clinical outcomes, the cost and accessibility of anti-CGRP therapies warrant consideration. Anti-CGRP mAbs, such as Erenumab, are costly, with an estimated annual expense of $6,900 in the United States. A report by the Pharmacy Benefit Management Institute (PBMI) highlighted that healthcare costs for patients with migraine are approximately $2,571 higher than those for patients without migraine. Despite these costs, awareness of CGRP inhibitors among patients remains low. Only one-third of migraine patients surveyed were aware of CGRP inhibitors as a treatment option, compared to higher awareness among healthcare plan sponsors [23]. Insurance coverage for these therapies also varies, with many plans favoring traditional preventive and acute treatments over specialty medications like CGRP inhibitors and Botox [23].

The findings of this meta-analysis are significant in the context of current clinical guidelines. The European Federation Headache Guideline supports anti-CGRP therapy for the preventive treatment of CM with MOH subgroup [11], aligning with the demonstrated efficacy and safety profiles observed in this study. Notably, the results suggest that both standard and high doses of anti-CGRP therapies yield comparable safety outcomes to placebo while achieving substantial efficacy, with at least three months of treatment. This supports the ethical commitment of healthcare professionals to “do no harm” while relieving patient suffering.

### Limitations

Despite the promising results, this meta-analysis has several important limitations. Cardiovascular adverse events were not comprehensively reported across the included studies, which should be a focus of future research. For Erenumab, although constipation is recognized as a significant adverse event, we were unable to evaluate this outcome due to its reporting in only a single study.

Another limitation lies in the initial analyses, where combining standard- and high-dose data introduced bias and heterogeneity, necessitating subgroup analyses to address these issues. While the subgroup analyses provided greater clarity on the efficacy and safety profiles of different dosages, moderate heterogeneity was observed for nasopharyngitis within the standard-dose group. A leave-one-out analysis was performed for this outcome, and with the exclusion of the “PROMISE 2” study, the results indicated a higher risk of nasopharyngitis in this subgroup. Finding may be attributed to the “Tepper 2024” study, a phase 4 trial that provided a more detailed investigation of adverse events associated with long-term treatment.

Additionally, the “PROMISE 2” study was assessed as having a high risk of bias due to selective reporting and subgroup analyses, while the “Yu 2023” study faced limitations related to missing outcome data. Sensitivity analyses were conducted to address these concerns, confirming the robustness of the primary findings; however, a residual risk of bias remains across the included studies.

Lastly, this meta-analysis included a limited number of studies, comprising only Eptinezumab and Erenumab. For Galcanezumab [30] and Fremanezumab [26, 28, 29] subgroup analysis of previous trials, all of the referenced studies claimed to address medication overuse headaches (MOH), but a closer examination of their inclusion criteria revealed that they broadly referred to “Medication Overuse,” a term that encompasses both MOH—requiring over three months of medication overuse for diagnosis—and Acute Medication Overuse (AMO), which lacks this temporal criterion. Our concern is that including these studies might allow us to analyze Fremanezumab and Galcanezumab, but at the cost of addressing the specific gap in the literature regarding their efficacy for MOH versus AMO. To maintain the integrity of our analysis and focus exclusively on MOH, we chose not to include these studies. Regarding the REGAN trial [30], we acknowledge that it included patients with MOH. However, we were unable to identify a separate dataset specifically for MOH within the study. This limitation prevented us from conducting a distinct analysis of MOH outcomes for this trial. This underscores the need for additional trials evaluating other anti-CGRP therapies in the chronic migraine (CM) and medication overuse headache (MOH) subgroups to better inform clinical practice.

## Conclusion

This systematic review and meta-analysis support the efficacy and safety of standard-to-high dose anti-CGRP therapy (Eptinezumab and Erenumab) in CM patients with MOH. Subgroup analyses demonstrate that high-dose therapy, compared to placebo, is notably more effective in achieving at least a 50% reduction in MMDs from baseline, without significant differences in safety outcomes. However, data on cardiovascular events and long-term less prevalent outcomes remain limited, as only one phase 4 RCT was included in this analysis. Therefore, further RCTs investigating long-term complications and safety profiles of these therapies are warranted. For now, our findings support the long-term use of both standard- and high-dose Eptinezumab and Erenumab in CM patents with MOH.

## Electronic supplementary material

Below is the link to the electronic supplementary material.

Supplementary Material 1

## Data Availability

No datasets were generated or analysed during the current study.
