# Supplementary material for: Safety, efficacy, and compliance of moderate-to-high dose eptinezumab and erenumab in chronic migraine patients with medication-overuse headache: an updated systematic review and meta-analysis
Source: J Headache Pain. 2025 May 6;26(1):99. doi: 10.1186/s10194-025-02047-7 (PMC12054139; doi:10.1186/s10194-025-02047-7)
Supplement: Supplementary file 1 — Supplementary Material 1 [file 10194_2025_2047_MOESM1_ESM.docx]

Table 1. Baseline characteristics of included studies

| Study | Design | Type of cGRPi | Patients  cGRPi/PBO | Male, %  cGRPi/PBO | Age ^†^,  cGRPi/  PBO | White, %  cGRPi/  PBO | Black, %  cGRPi/PBO | Baseline MMDs ^†^,  days  cGRPi/PBO | Baseline MHD ^†^, days  cGRPi/PBO | Baseline simple analgesic overuse, n (%)  cGRPi/PBO | Baseline triptan/ergot/opioid overuse, n (%) cGRPi/PBO |
| --- | --- | --- | --- | --- | --- | --- | --- | --- | --- | --- | --- |
| Diener 2020 | RCT | Eptinezumab  100 mg | 139/145 | 12.2/13.8 | 41.5 (11.36)/40.7 (10.86) | 94.4/  91.7 | 4.9/  6.2 | 16.7 (4.58)/  16.7 (4.43) | 20.7 (3.00)/  20.7 (3.00) | 26 (18.7)/  30 (20.7) | 83 (59.7)/  88 (60.7) |
| Tepper 2024 | RCT | Erenumab  140 mg | 194/194 | 16.4/19.6 | 43.5 (12.0)/  44.4 (12.6) | 90.2/  94.8 | 1.53/1 | 18.5 (4.6)/  18.6 (4.6) | 20.7 (3.8)/  20.8 (3.9) | 12 (6.2)/  19 (9.8) | 140 (71.8)/  128 (66) |
| Yu 2023 | RCT | Eptinezumab  100 mg | 93/100 | 26.7/18 | 43.5 (4.9)/  44.5 (4.9) | N/A | N/A | 19.5 (3.6)/  19.7 (3.8) | 20.6 (2.9)/  20.9 (3.3) | N/A | N/A |

RCT: randomized controlled trial; PBO: Placebo; cGRPi: anti-Calcitonin Gene-Related Peptide monoclonal antibodies; ^†^mean (SD); MMD: Monthly Migraine Days; MHDs: Monthly Headache Days; n: number of events.

Definitions

1. MMDs: The total number of days in a calendar month during which a patient experiences a migraine attack meeting diagnostic criteria (e.g., pain severity, duration, associated symptoms such as nausea or sensitivity to light/sound).
2. MOH remission; ICHD-3 Criteria: Headache days reduced to <15 days/month after discontinuing the overused medication for at least 2 months.
3. TESAEs: A serious adverse event is defined as a medical event linked to a drug, device, or intervention that results in death, a life-threatening condition, hospitalization, significant disability, a congenital anomaly, or requires intervention to prevent permanent harm.
4. MHDs: the total number of days in a calendar month where a patient experiences a headache, regardless of whether the headache fulfills the specific criteria for a migraine.

**Supplementary Table 1. Critical appraisal of individual studies according to the Cochrane Collaboration’s tool for assessing risk of bias in randomized trials.**

| **Study** | **Bias from randomization process** | **Bias due to deviations from intended interventions** | **Bias due to missing outcome data** | **Bias in measurement of the outcomes** | **Bias in selection of the reported result** | **Overall risk of bias** |
| --- | --- | --- | --- | --- | --- | --- |
| Tepper-2024 | Some concerns | low | low | low | low | Some concerns |
| Yu-2023 | Low | Some concerns | low* | low | low | Some concerns |
| Promise 2-2020 | Low | Some concerns | low | low | High | High |

*For Bias due to missing outcome data, Yu 2023 was subjected as three patients were excluded from the data analysis set. Therefore, we did sensitivity analyses for outcome of interest (50 percent reduction from baseline in MMDs). Assume the best-case scenario, and the worst-case scenario for above outcome (Supplementary 1).

Risk of Bias for randomized controlled trials (RoB) for Yu 2023 with sensitivity analysis, as there are missing data outcomes for 3 patients who were excluded from the study.

**Previous result.**


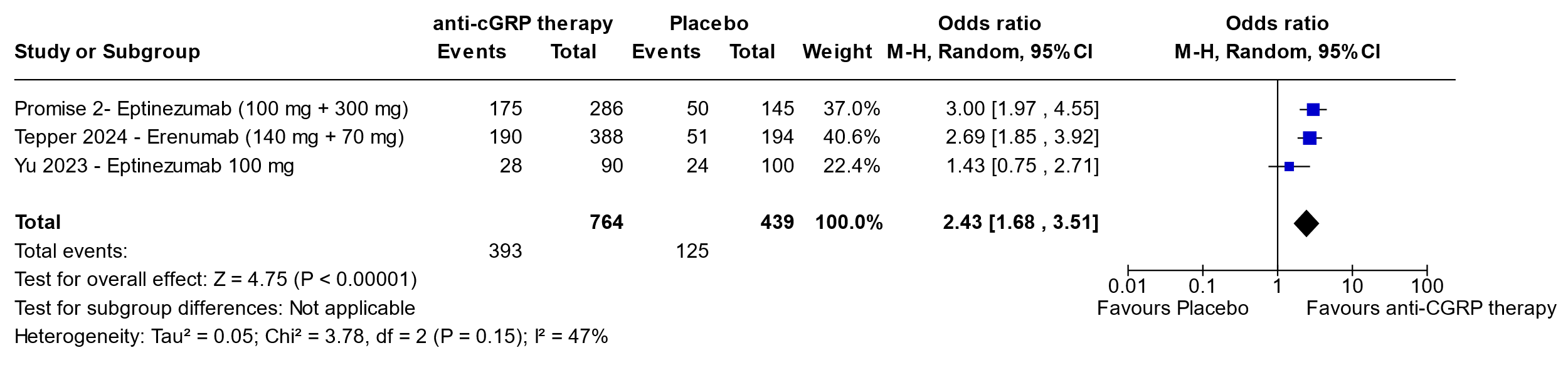
**Figure 2A.At least a 50 percent reduction in Monthly Migraine Days (MMDs) from baseline, with at least 12 weeks of treatment.**

**We assume the best case scenario in which all patients achieved at least 50 percent reduction in MMDs from baseline; 31 patients from Yu 2023 would yield the outcome of interest, instead of 28 ones. Image below.**

**
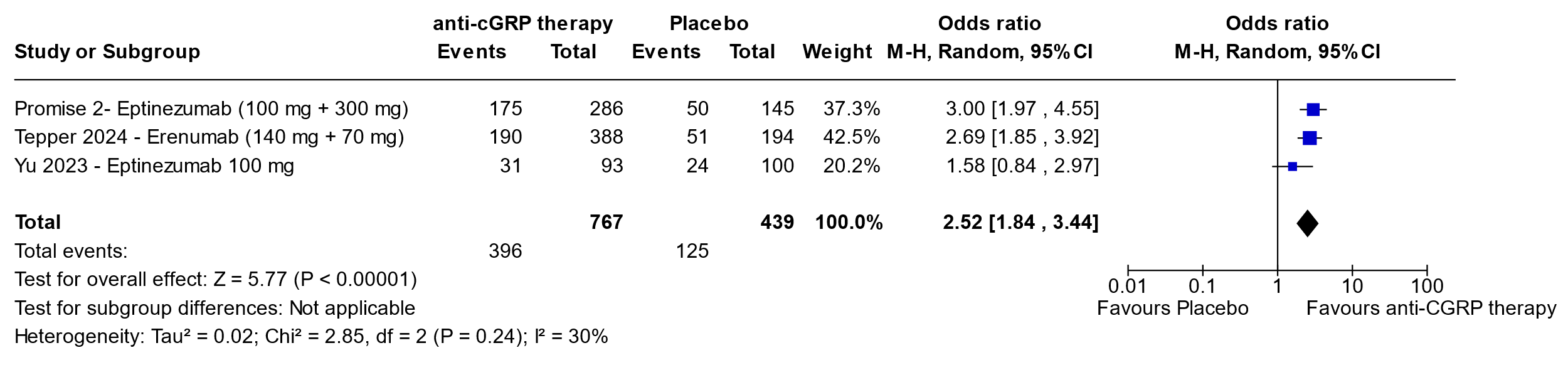
**

**Figure 2A:** Patients who underwent anti-CGRP therapy were more than twice as likely to achieve at least a 50% reduction in monthly migraine days (MMDs) compared to the placebo group after at least 12 weeks (p < 0.05; OR = 2.52 [1.84, 3.44]).

**In the worst-case scenario for TESAEs, we assume that all three patients did not develop this outcome to re-evaluate the efficacy, which yields the number of patients with the event same as previous analysis in the anti-CGRP therapy group, but total number of patients is up to 93.**


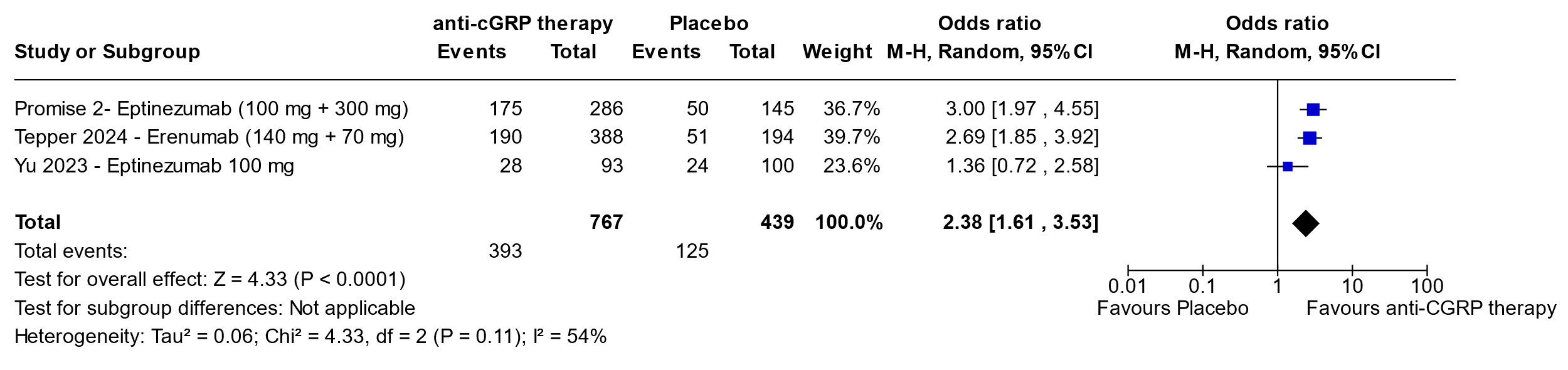


**Figure 2A:** Patients who underwent anti-CGRP therapy were more than twice as likely to achieve at least a 50% reduction in monthly migraine days (MMDs) compared to the placebo group after at least 12 weeks (p < 0.05; OR = 2.38 [1.61, 3.53]).

**Conclusion RoB for Yu 2023:** Compared to our previous result, the OR increased up to 2.5 in the best case scenario; down to 2.38 for the worst case scenario. However, this result would not influence significantly our finding as both are well above twice (under triple) the likelihood of achievement of at least a 50 percent reduction in MMDs. In conclusion, we conclude that the RoB due to missing data is relatively low for Yu 2023.

**Forest plots**

- **Overall Anti-CGRP Therapy Efficacy (combined moderate and high dose therapy)**

**Figure 2D.Adverse Events Reported by Participants: Nasopharyngitis.**

**
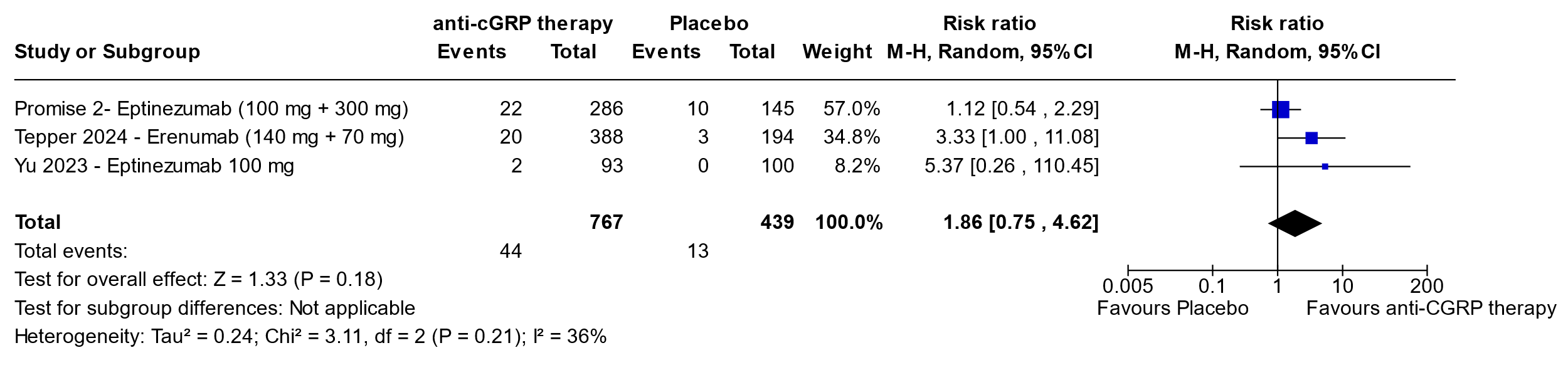
**

**Figure 2D:** There were no significant differences between the two groups—anti-CGRP therapy and placebo—in the risk of nasopharyngitis, as the p-value (p = 0.18) is greater than 0.05. The confidence interval includes the null value of 1 (RR = 1.86 [0.75, 4.62]).

**Figure 2E.Adverse Events Reported by Participants: Upper Respiratory Infection.**


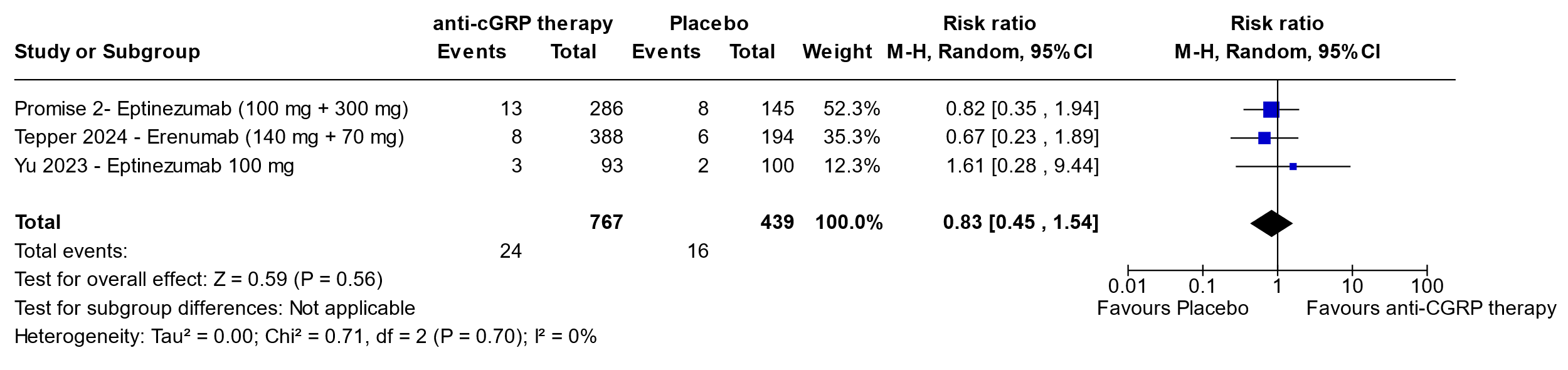


**Figure 2E:** There were no significant differences between the two groups—anti-CGRP therapy and placebo—in the risk of upper respiratory infection, as the p-value (p = 0.56) is greater than 0.05. The confidence interval includes the null value of 1 (RR = 0.83 [0.45, 1.54]).

**Figure 2F. Treatment-Emergent Adverse Events (TEAEs) Leading To Drug Discontinuation or Interruption.**


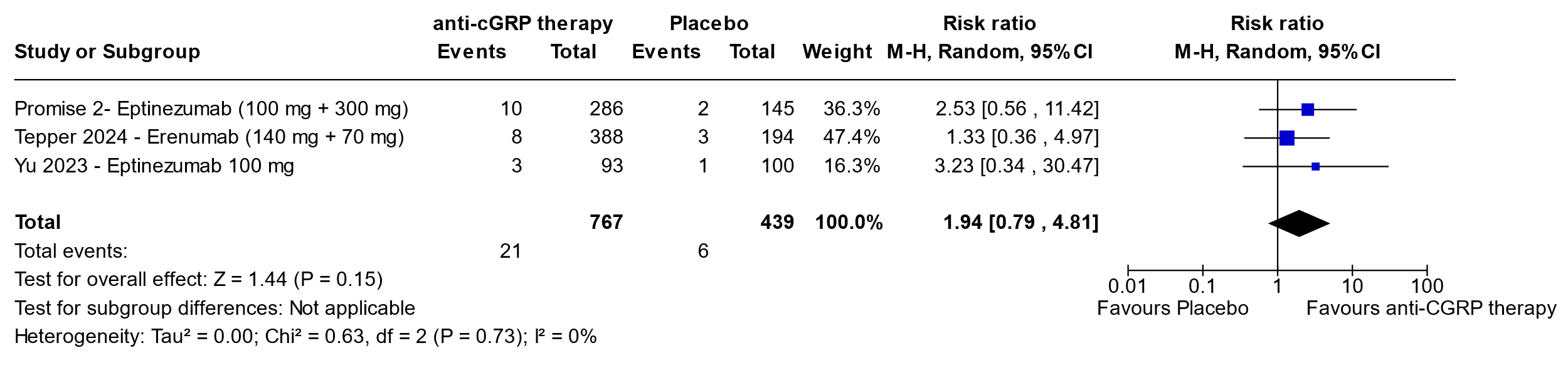


**Figure 2F:** There were no significant differences between the two groups—anti-CGRP therapy and placebo—in the risk of treatment-emergent adverse events (TAEs) leading to drug discontinuation or interruption, as the p-value (p = 0.15) is greater than 0.05. The confidence interval includes the null value of 1 (RR = 1.94 [0.79, 4.81]).

- **Subgroup Analysis: Eptinezumab 100 mg, and Erenumab 70 mg (Moderate Dose)**

**Figure 3A. At least 50 percent reduction in Monthly Migraine Days (MMDs) from baseline, with at least 12 weeks of treatment**

**
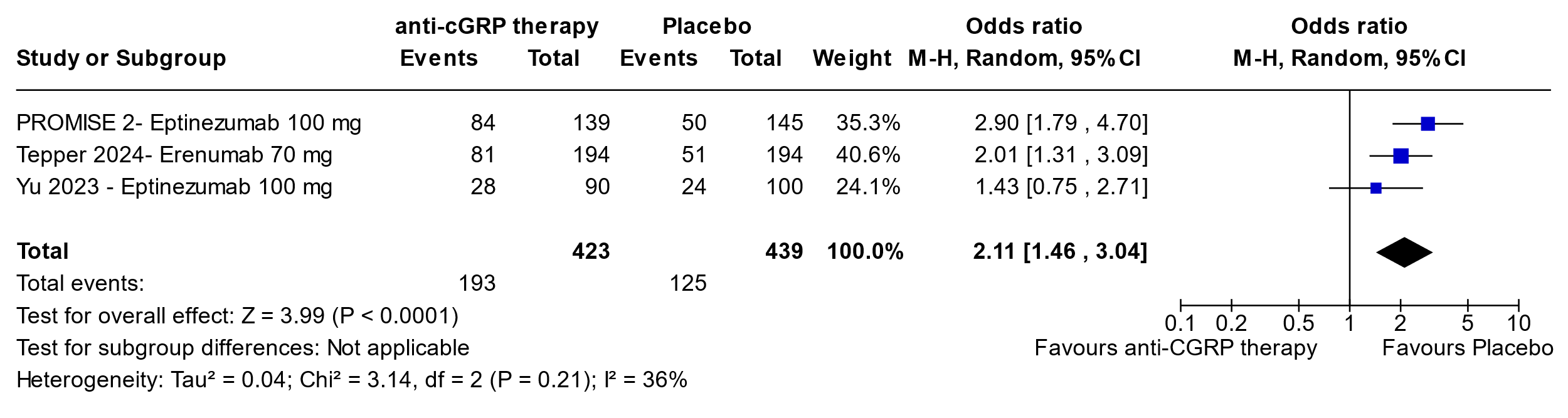
**

**Figure 3A:** Patients who underwent anti-CGRP therapy were more than twice as likely to achieve at least a 50% reduction in monthly migraine days (MMDs) compared to the placebo group after at least 12 weeks (p < 0.00001; OR = 2.11 [1.46, 3.04]).

**Figure 3B. MOH remission at 6 months.**

**
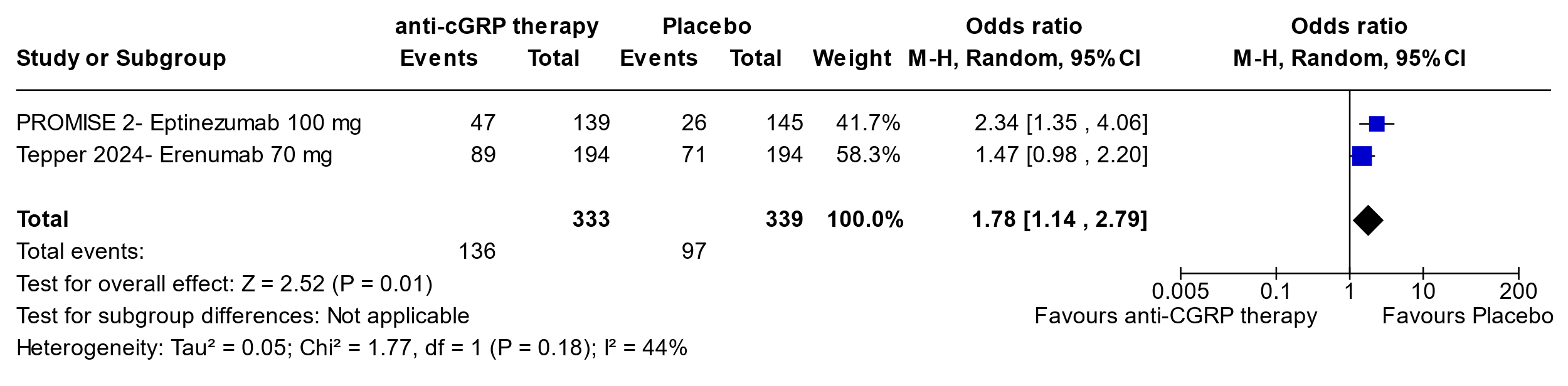
**

**Figure 3B:** Patients who underwent anti-CGRP therapy were approximately twice as likely to achieve MOH remission at 6 months compared to the placebo group (p = 0.01; OR = 1.78 [1.14, 2.79]).

**Figure 3C. Treatment Emergent Serious Adverse Events (TESAEs)**

**
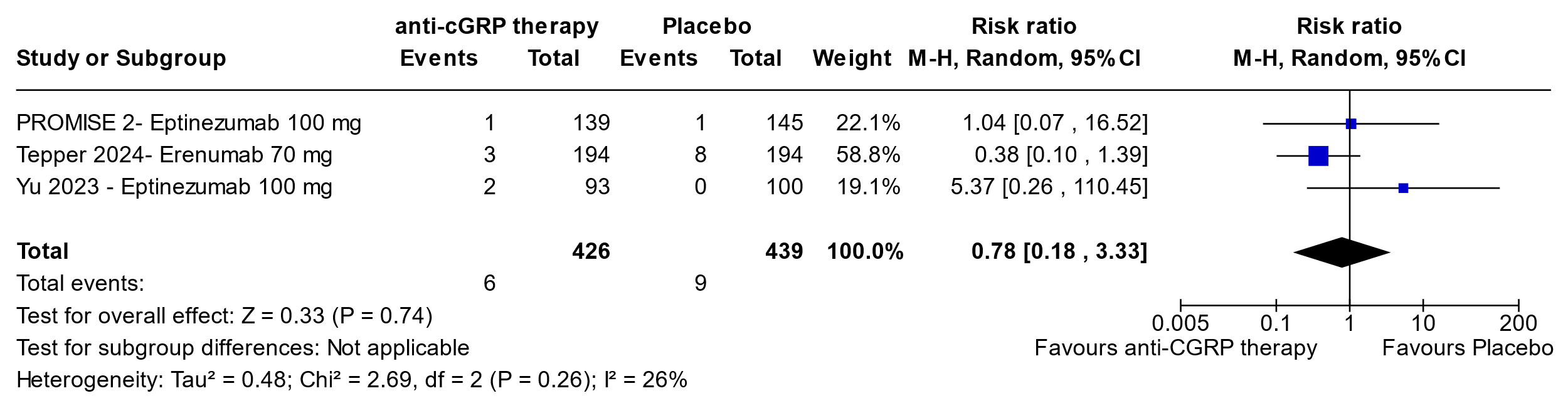
**

**Figure 3C:** There were no significant differences between the two groups—standard dose and placebo—in the risk of treatment-emergent serious adverse events (TAEs), as the p-value (p = 0.74) is greater than 0.05. The confidence interval includes the null value of 1 (RR = 0.78 [0.18, 3.33]).

**Figure 3D. Adverse Events Reported by Participants; Nasopharyngitis**


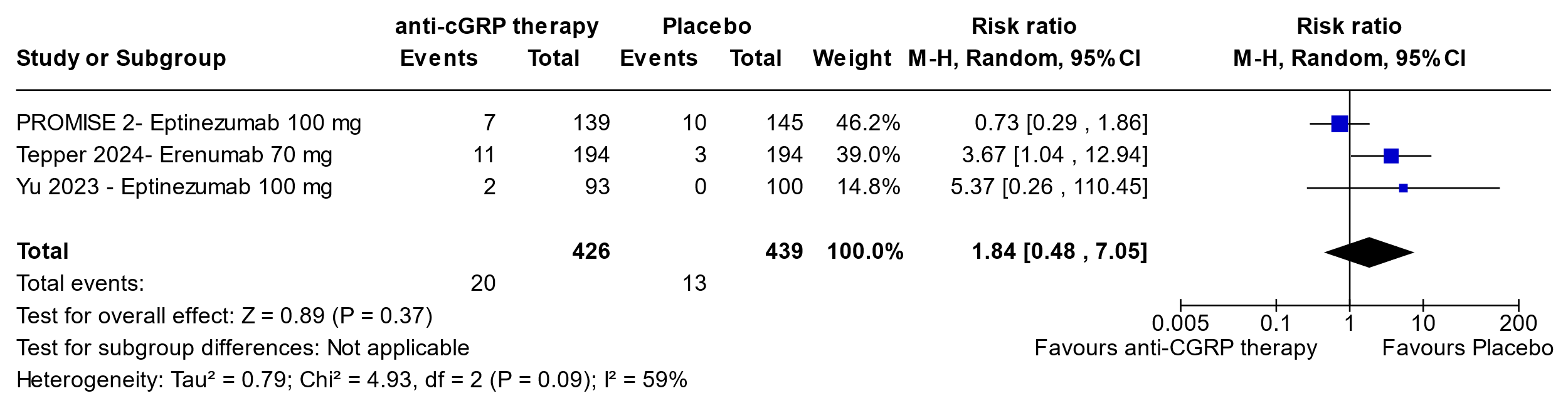


**Figure 3D:** There were no significant differences between the two groups—standard dose and placebo—in the risk of nasopharyngitis, as the p-value (p = 0.37) is greater than 0.05. The confidence interval includes the null value of 1 (RR = 1.84 [0.48, 7.05]).

**Figure 3D. Adverse Events Reported by Participants; Nasopharyngitis; leave-one-out analysis (PROMISE 2).**

**
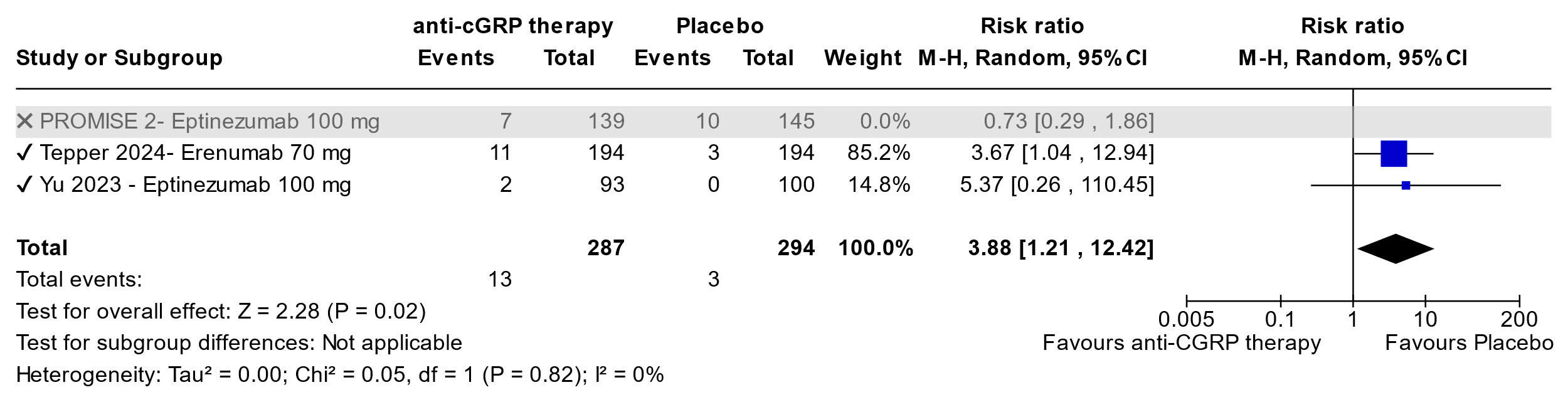
**

**Figure 3E. Adverse Events Reported by Participants; Upper Respiratory Infection**

**
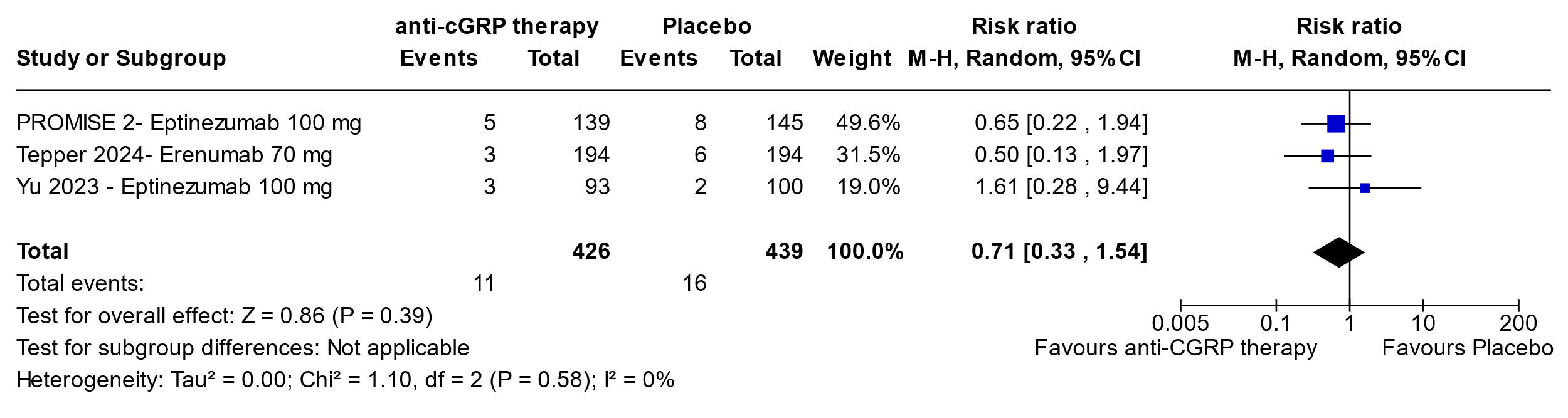
**

**Figure 3E:** There were no significant differences between the two groups—standard dose and placebo—in the risk of upper respiratory infection, as the p-value (p = 0.39) is greater than 0.05. The confidence interval includes the null value of 1 (RR = 0.71 [0.33, 1.54]).

**Figure 3F. Treatment Emergent Adverse Events (TEAEs) Leading To Drug Discontinuation or Interruption**

**
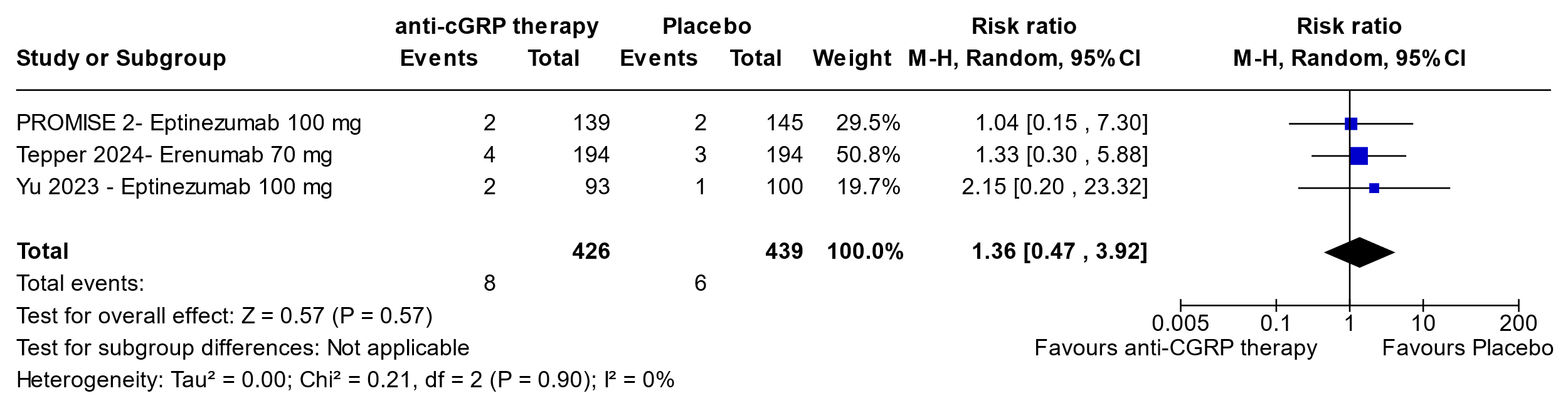
**

**Figure 3F:** There were no significant differences between the two groups—standard dose and placebo—in the risk of treatment-emergent adverse events (TAEs) leading to drug discontinuation or interruption, as the p-value (p = 0.57) is greater than 0.05. The confidence interval includes the null value of 1 (RR = 1.36 [0.47, 3.92]).

- **Subgroup Analysis: High-Dose Anti-CGRP Therapy (Eptinezumab 300 mg, Erenumab 140 mg)**

**Figure 4D. Adverse Events Reported by Participants; Nasopharyngitis.**


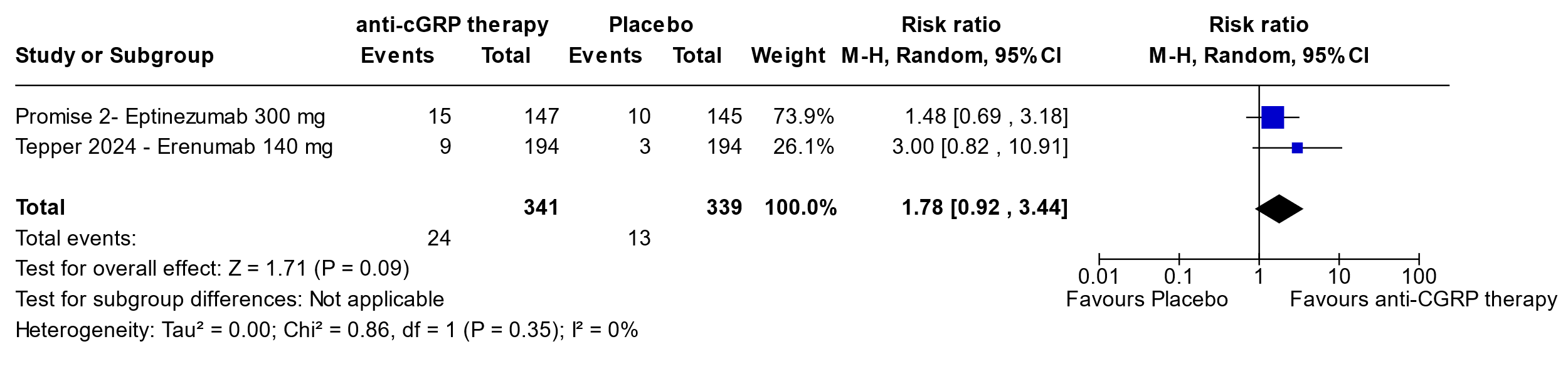


**Figure 4D:** There were no significant differences between the two groups—high dose therapy and placebo—in the risk of nasopharyngitis, as the p-value (p = 0.09) is greater than 0.05. The confidence interval includes the null value of 1 (RR = 1.78 [0.92, 3.44]).

**Figure 4E. Adverse Events Reported by Participants; Upper Respiratory Infection.**


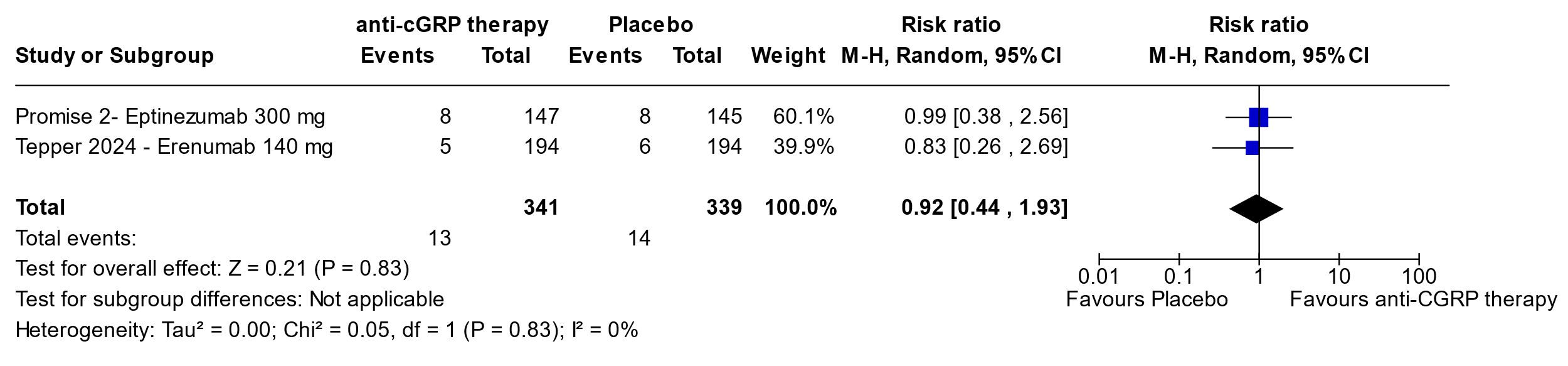


**Figure 4E:** There were no significant differences between the two groups—high-dose therapy and placebo—in the risk of upper respiratory infection, as the p-value (p = 0.83) is greater than 0.05. The confidence interval includes the null value of 1 (RR = 0.92 [0.44, 1.93]).

**Figure 4F. Treatment Emergent Adverse Events (TEAEs) Leading To Drug Discontinuation or Interruption.**


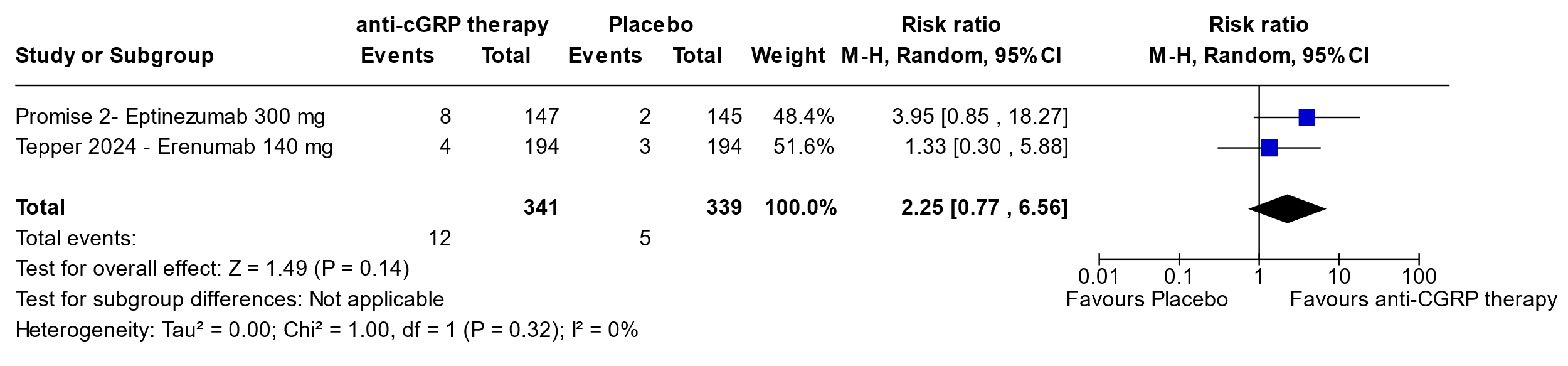


**Figure 4F:** There were no significant differences between the two groups—high-dose anti-CGRP therapy and placebo—in the risk of treatment-emergent adverse events (TAEs) leading to drug discontinuation or interruption, as the p-value (p = 0.14) is greater than 0.05. The confidence interval includes the null value of 1 (RR = 2.25 [0.77, 6.56]).

- **MOH remission reanalyzed using acute headache medication subgroup sample sizes in PROMISE 2 (n=200 active, n=96 placebo), ; results remained consistent across high- and standard-dose subgroups.**

**Figure 5A. Overall Anti-CGRP Therapy Efficacy (combined moderate and high dose therapy)**

**
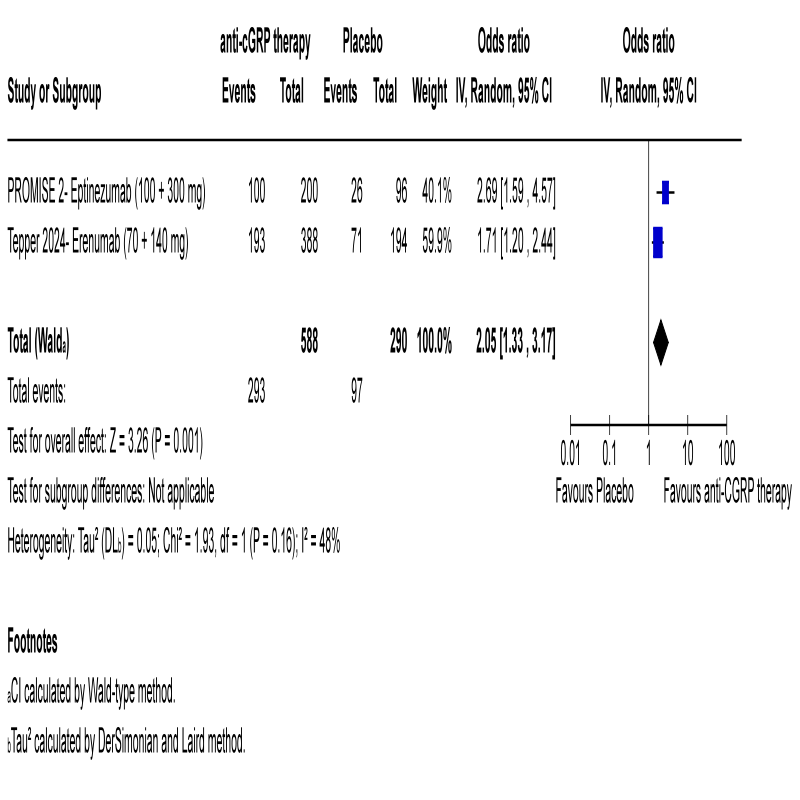
**

**Figure 5A:** Patients who underwent anti-CGRP therapy were approximately twice as likely to achieve MOH remission at 6 months compared to the placebo group (p = 0.001; OR = 2.05 [1.33, 3.17])

**Figure 5B: Subgroup Analysis: High-Dose Anti-CGRP Therapy (Eptinezumab 300 mg, Erenumab 140 mg)**

**
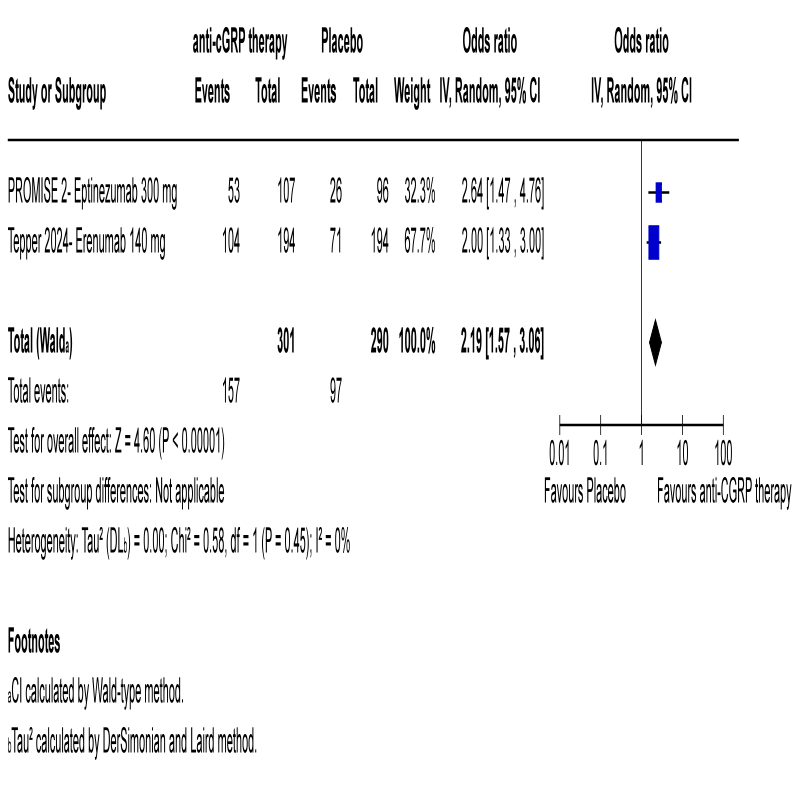
**

**Figure 5B:** Patients who underwent anti-CGRP therapy were approximately twice as likely to achieve MOH remission at 6 months compared to the placebo group (p < 0.00001; OR = 2.19 [1.57, 3.06])

**Figure 5C: Subgroup Analysis: Eptinezumab 100 mg, and Erenumab 70 mg (Moderate Dose)**

**
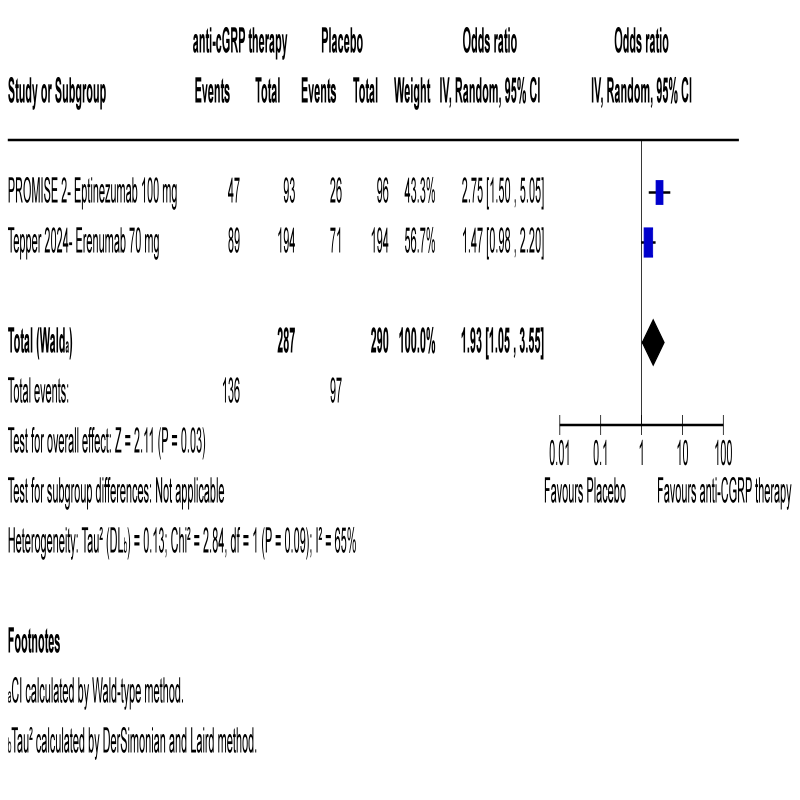
**

**Figure 5C:** Patients who underwent anti-CGRP therapy were approximately twice as likely to achieve MOH remission at 6 months compared to the placebo group (p = 0.03; OR = 1.93 [1.05, 3.55])
